# A Novel Imaging Protocol for Investigating *Arabidopsis thaliana* Siliques and Seeds Using X-rays

**DOI:** 10.21769/BioProtoc.4839

**Published:** 2023-10-05

**Authors:** Brylie A. Ritchie, Theodore A. Uyeno, Daniel Rincon F. Diaz, Ansul Lokdarshi

**Affiliations:** Department of Biology, Valdosta State University, Valdosta, GA, USA

**Keywords:** X-rays, Plant development and morphogenesis, *Arabidopsis thaliana* siliques and seeds

## Abstract

Understanding silique and seed morphology is essential to developmental biology. Arabidopsis thaliana is one of the best-studied plant models for understanding the genetic determinants of seed count and size. However, the small size of its seeds, and their encasement in a pod known as silique, makes investigating their numbers and morphology both time consuming and tedious. Researchers often report bulk seed weights as an indicator of average seed size, but this overlooks individual seed details. Removal of the seeds and subsequent image analysis is possible, but automated counts are often impossible due to seed pigmentation and shadowing. Traditional ways of analyzing seed count and size, without their removal from the silique, involve lengthy histological processing (24–48 h) and the use of toxic organic solvents. We developed a method that is non-invasive, requires minimal sample processing, and obtains data in a short period of time (1–2 h). This method uses a custom X-ray imaging system to visualize Arabidopsis siliques at different stages of their growth. We show that this process can be successfully used to analyze the overall topology of Arabidopsis siliques and seed size and count. This new method can be easily adapted for other plant models.

Key features

• No requirement for organic solvents for imaging siliques.

• Easy image capture and rapid turnaround time for obtaining data.

• Protocol may be easily adapted for other plant models.

**Graphical overview**


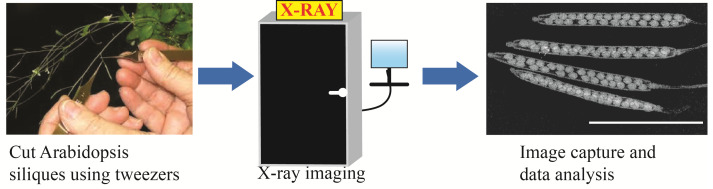


***Arabidopsis* siliques using the Inspex 20i X-ray machine**

## Background

Seed development is critical for the survival of all flowering plants (Jardinaud and Petitprez, 2003). Of the current plant models, *Arabidopsis thaliana*, with its convenient seed pod (silique), continues to be the most widely used tool for studying fruit/seed development and morphogenesis (Bates et al., 2013; Kao and Nodine, 2021). *Arabidopsis* siliques contain rigid cell walls and are pigmented by chlorophyll, which makes analyses of seed morphology, anatomy, and ultrastructure challenging (Hedhly et al., 2018; Attuluri et al., 2022). To overcome these difficulties, conventional methods employ different types of fixatives (e.g., Acryloyl-X, FPA50) and optical clearing reagents (e.g., chloral hydrate, 2,2’-thiodiethanol, sodium dodecyl sulfate) for whole-mount microscopy (Hedhly et al., 2018; Attuluri et al., 2022). Despite their pervasive use, these methods are tedious, as these clearing procedures have specific protocols that involve the use of harmful organic solvents and lengthy (> 24 h) durations (Attuluri et al., 2022). Hence, there is a growing demand for non-invasive techniques that can provide high-quality digital data with shorter turnaround times and avoid the use of toxic organic solvents. In the work presented here, we describe a new, non-invasive technique for visualizing *Arabidopsis* siliques with seeds at different stages of their growth using a custom X-ray imaging system. While X-ray microscopy has been used for imaging flower development in *Arabidopsis* (Prunet and Duncan, 2020), studies of seed and silique development continue to rely mostly on conventional histological methods. We show that with minimal sample processing, without any use of toxic organic solvents, high-quality images can be captured with our custom X-ray imaging system (in 1–2 h) for downstream processing and data analyses. Statistical tests using one-way ANOVA and frequency distribution show high reproducibility and consistency of seed count and seed diameter in dried *Arabidopsis* siliques across three independent experiments. Taken together, our easy-to-use X-ray system serves as a new imaging tool for visualizing *Arabidopsis* siliques and seeds and, with some modification, can be adapted for other plant models.

## Materials and reagents

**Biological materials**

*Arabidopsis thaliana* ecotype Columbia (Col_0) plant at midflowering, complete flowering, and seed harvest stage (Boyes et al., 2001).

**Laboratory supplies**

1. Fine precision medium tipped tweezers/forceps (Fisher Scientific, catalog number: 12-000-157)
2. Petri dish (Fisher Scientific, catalog number: FB0875712)
3. Permanent ink markers
4. Paper towels (Fisher Scientific, catalog number: 19-040-898)
5. Tape (Fisher Scientific, catalog number: 15949)
6. X-ray source: Kevex PXS10-16W MicroFocus system
7. Boss LS-1416 CO_2_ laser cutter

## Equipment

1. Inspex 20i X-ray machine
  For custom manufacturing of the X-ray imaging cabinet, details are provided in Figure 1. The Inspex 20i X-ray machine was assembled by Kodex Inc. (Nutley, NJ).
  **Figure 1.**
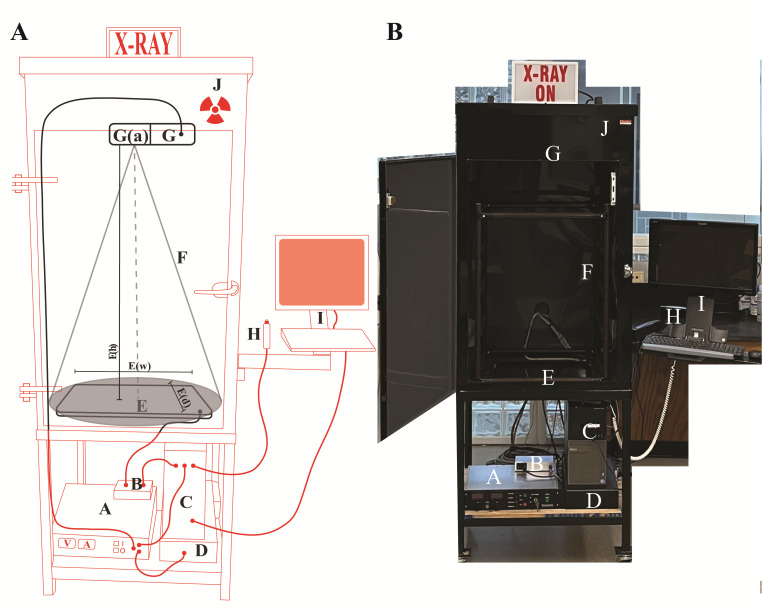
Schematics of the X-ray imaging setup. (A) Cartoon sketch of the Inspex 20i X-ray machine with all the accessories. The custom steel cabinet (J) measures 64.77 cm (depth) × 127 cm (height) × 78.74 cm (width) and is lined with a lead sheet to conform to BRH 21 CFR 1020.40 and FDA 21 CFR 1020.40, in accordance with Georgia State X-ray radiation regulations. The X-ray source (G) is a Kevex PXS10-16W MicroFocus system that produces a downward facing cone-beam so that shadows can be detected on the Kodex LTX-1717 digital detection panel (E) located at the bottom of the cabinet. This amorphous silicon flat panel detector has a cesium iodide scintillator in the outer layer that allows for the conversion of X-rays to light, which is then digitized by the photodiode layer below for transmission to the computer for display on the screen. The microfocus system allows focused images to be enlarged or reduced by varying the specimen height between the source and detector (closer to the source results in a magnified image). Placing the specimen as close as possible to the source (G­(a)) results in a field of view of approximately 9.5 mm^2^. The field of view adjacent to the detector (E) is approximately 45 times greater at 431.8 mm^2^ (the detector is 17 × 17 inches). Components external to the cabinet (red): (A) Power supply for the X-ray source; (B) Power supply and data transfer unit for the detector; (C) Computer to render and save images; (D) Uninterruptable power supply and surge suppressor for the X-ray machine. Components inside the cabinet (black): (E) The Kodex LTX-1717 digital detection panel, E(w) width 44.45 cm, E(d) depth 44.45 cm, E(h) distance between detector and source 104.14 cm; (F) The cone beam path of the X-ray (cone of illumination, 53°); (G) The Kevex PXS10 MicroFocus X-ray source (maximum settings of 80 kV, 0.100 mA); (H) Image collection shutter trigger; (I) Touchscreen monitor and keyboard for image viewing. (Average settings for this study: Silique specimens in this study were placed on a low-density plastic platform suspended 98.4 cm above the detector, and the source was operated at 40 kV and 0.09 Amps). (B) Picture of the X-ray machine with the front door open.
2. Scale for the measurement of siliques/seeds using the Inspex 20i X-ray machine
  We used an X-ray semi-transparent scale that was cut from cardstock (weight 90 Lb) using a Boss LS-1416 CO_2_ laser cutter (70 W). We did this because the printed hashmarks on solid plastic or metal rulers are not visible in X-ray images. We designed the scale using the Adobe Illustrator vector drawing package. Only the millimeter hashmarks and the external contour of the scale were cut; the millimeter number markings were not. The kerf width of the laser cutter (aka. hashmark line width) measured an average of 0.152 mm. This was wider than we had hoped, but the centers of the marks were extremely accurate. The dimensional accuracy of the laser cutter was listed as being ± 0.0127 mm, and this high accuracy was confirmed using multiple micrometer measurements from the hashmark centers. Since the scale bars and their hashmarks could be seen beside the seeds and siliques, they proved a simple, economical, and reproducible method of calibrating X-ray image dimensions.

## Software and datasets

1. VetView Console: All-In-One Workstation Software is used for image acquisition, processing, and editing.
2. DaVinci Resolve 11: Calibration software, run as a script, to dark calibrate data read from the X-ray detector while the source is not providing illumination.
3. FIJI ImageJ Build 2.13.1: This image processing program was used to count and measure diameters of the seeds within the silique.
4. GraphPad Prism Version 9.4.1: Statistical analyses and production of graphs.

## Procedure

Please see Video 1 for sections A, B, and C detailing the procedure.

**Video 1.**
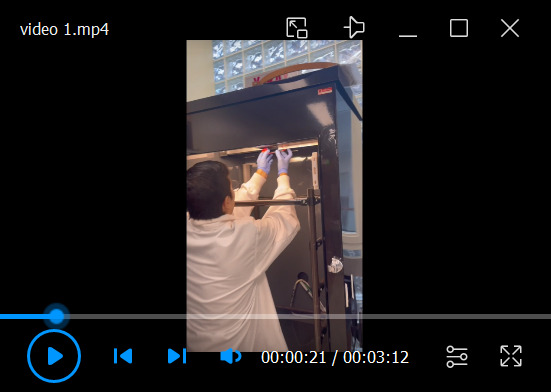
Video tutorial showing the sample preparation and X-ray imaging procedure

- **Setting up low-density plastic platform in the Inspex 20i X-ray machine for sample tray (see Figures 1, 2 for details)**
  **Figure 2.**
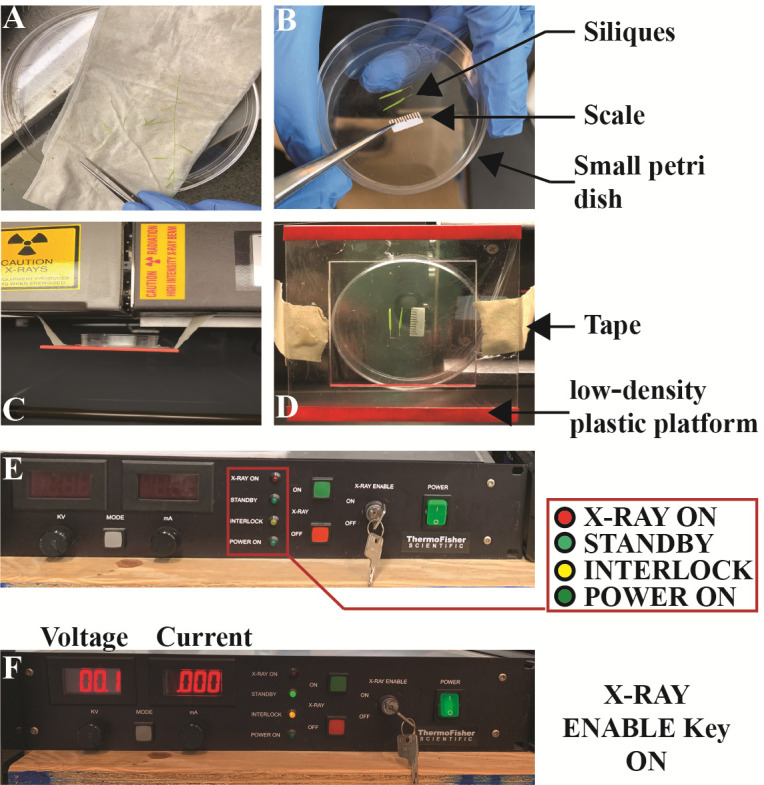
Sample loading and readiness of the Inspex 20i X-ray machine. (A) Large Petri dish with silique samples on damp paper towel. (B) Small Petri dish with two silique samples and scale placement using metal tweezer. (C, D) Lateral (C) and Overhead (D) view of the low-density platform held by tape at both ends. Also shown is the Petri dish carrying silique samples and scale shown in panel B. (E, F) Power supply for the X-ray source showing X-ray key enabled in panel F.
  1. Open the front door of the X-ray machine by pulling the metal latch located at the center-right of cabinet J.
  2. Tape the low-density plastic platform ~3.5 cm underneath the source G. We found that the highest contrast images were made when specimens were imaged on a platform made of low-density polyethylene plastic films. This is because these films are radiotransparent relative to the siliques, seeds, and paper calibration scales. However, many other plastics such as the polystyrenes and polycarbonates used in plastic Petri dishes can also be used with good results.
  3. Close the front door by pushing the metal latch handle until the click sound confirming the interlock system has engaged is heard.
- **Calibration of Inspex 20i X-ray machine (see Figure 1 and Figure 2E–2F for details)**
  1. Press the power switch located on control panel A for switching the control panel A ON.
  2. Turn ON the control panel B using the power switch.
  3. Turn ON panel C to start the desktop by pressing the power button ON.
  4. On the control panel A, turn the metal key counterclockwise to position ON and press the green button on panel A.
*Note: The following lights should be visible now: Red color for X-ray ON, yellow for interlock, and green flashing indicating the main power ON. See Figure 2 for details.*
  5. Wait for the X-ray tube unit to warm up for ~10–15 min.
*Note: As part of the warm-up sequence, the X-ray sign above the steel cabinet J will illuminate and the numbers on the control panel A (Figure 2F) will show a gradual increase in voltage and current (Amps).*
  6. Full system readiness is marked by illumination of the green light next to the standby on panel A, the readings for volts and amps set at 0, and the X-ray sign OFF (Figure 2F).
  7. Open the front door (see step B1) and ensure that the overhead lights inside cabinet J are not illuminated. If the light is off, then close the front door and proceed to the next step.
  8. Double click on the dark calibration tab located on the desktop to run dark calibration.
*Notes:*
    - *This step is required for best image contrast.*
    - *As part of this step, a pop-up window will appear on the desktop screen and then disappear, indicating step completion.*
  9. Repeat step B8.
- **Harvesting *Arabidopsis* siliques and imaging procedure with Inspex 20i X-ray machine (see Figures 1 and 2 for details)**
  We used standard methods for growing *Arabidopsis* plants on soil. This step is a standard procedure in plant biology and various resources are commonly available.
  1. Dampen one paper towel with deionized water and place it into a large Petri dish.
*Note: The entire paper towel should be wet, then squeeze out the extra deionized water.*
  2. For harvesting siliques of the desired growth stage (Boyes et al., 2001), use tweezers to gently pluck one silique at a time and place it into a clear Petri dish.
*Note: Gentle care is highly recommended for handling dried siliques as they tend to split and release the seeds.*
  3. Retrieve siliques, with pedicle attached, using fine-tipped tweezers and place on the damp paper towel until ready for next step.
    *Note: Siliques can stay for a maximum of 1–2 min only on the damp paper towel, as longer wait times may trigger imbibition in dry siliques*.
  4. Transfer one silique into the base of a small clean Petri dish.
*Note: A maximum of 5–6 siliques can be placed side-by-side to improve throughput.*
  5. Place the 1 cm scale bar next to the siliques in the small Petri dish.
  6. Open the front door of cabinet J and place the small Petri dish onto the low-density plastic platform.
*Note: A few trial runs may be required to adjust sample positioning, focus, and overall image quality.*
  7. Close the front door and open the VetView console program on the desktop.
  8. Click “new sample” tab and label the sample using the sample ID and sample name boxes.
  9. Click capture and wait for a pop-up window.
*Note: Ensure that the new window is set to the whole sample and then click capture.*
  10. Click the green ON button on control panel A.
  11. On the control panel A, set volts to 40.0 and amps to 0.09 using the dial knobs below the number screen.
  12. Click the handheld X-ray button H.
  13. As the VetView program displays “image acquiring”, click the red button on control panel A to turn the X-ray beam OFF.
*Note: This step ensures an extended life for the X-ray bulb. Leave X-ray beam on for no longer than one minute after image is acquired.*
  14. Image will appear on the desktop screen and will show a white square placed around the center of the image.
*Note: The white square is a tool to assist with centering the image and cropping unnecessary blank space.*
  15. Ensure your sample and scale bar are within the marquee by left clicking on the marquee edge and dragging it to center your samples inside the marquee frame.
*Note: The shape of the marquee can be adjusted by dragging either the corners or the sides.*
  16. Select “accept image” for downstream processing.
*Note: For reacquiring an image, select “reject image”. Repeat step D5 by adjusting the small Petri dish with the silique samples. Click edit protocol on the desktop screen and start from step D9.*
  17. After accepting the desired image, the VetView console program will reconfigure to the home page.
  18. Select the name provided for the sample (step D7) and click open study.
*Note: This will allow you to right-click to adjust the contrast.*
  19. Click the “save” tab to save the image file and close study to return to the home page of the VetView console program.
*Note: Check saved images in the destination folder before proceeding to another round of imaging.*
  20. Remove the small Petri dish with silique samples from the X-ray machine by opening the front door of cabinet J.
  21. Repeat steps D3–D18 for acquiring additional images.
  22. When the experiment is finished, turn off the overhead lights in cabinet J and power down the X-ray machine by switching off the desktop (panel C) and control panels A and B.
*Note: Powering OFF sequence is in the opposite order as discussed in section C for powering ON.*

## Data analysis

**Results**

**Figure 3.**
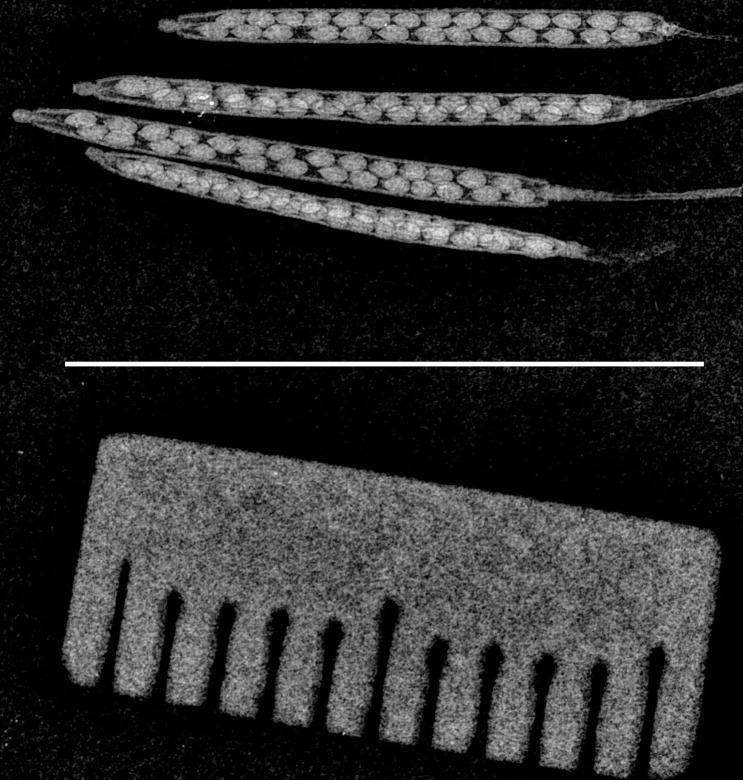
Image of siliques with scale bar. Representative image of siliques at maturity stage (dry) with the scale at the bottom. White color scale bar (1 cm) is shown in the center of the image.

**Figure 4.**
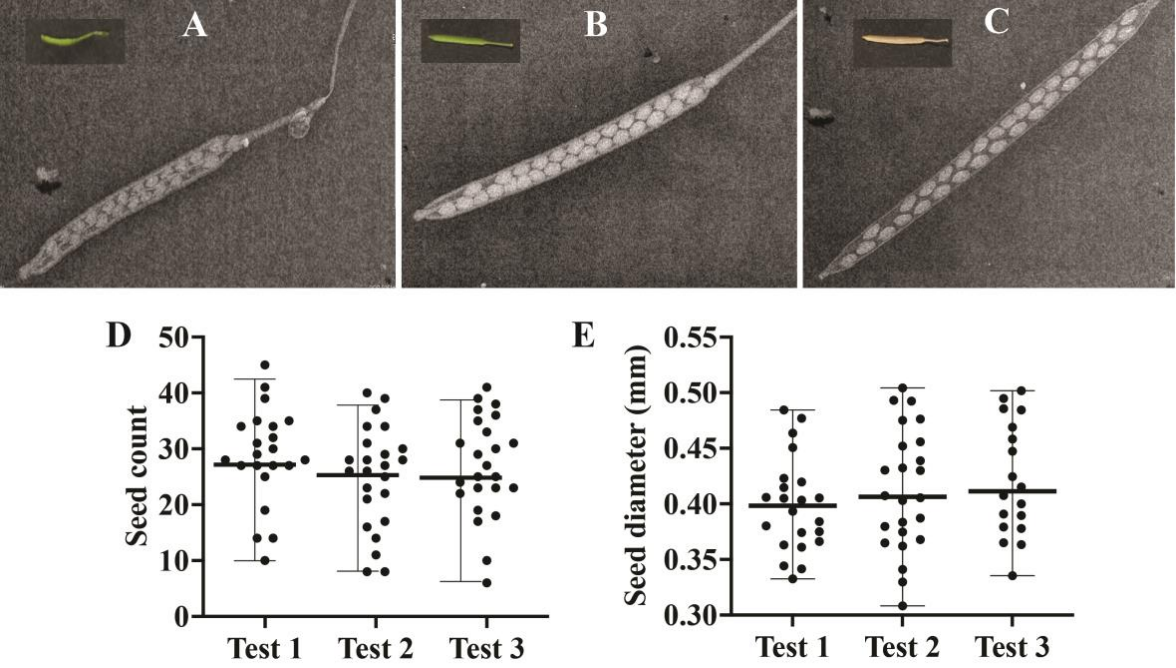
Silique and seed imaging using Inspex 20i X-ray machine and statistical tests for reproducibility. Representative image of silique at (A) young, (B) mature, and (C) dry stages of maturity captured by the Inspex 20i X-ray machine. Inset in each panel shows an example image of the silique at different stages of maturity. (D) Total seed count (y-axis) and (E) seed diameter (y-axis) in millimeters (mm) of dry siliques from three independent experiments (x-axis - Test 1, 2, 3). Center bold horizontal line within each test represents the median (panel D: 26–28.5; panel E: 0.39–0.41), while the range of distribution is shown with horizontal small edges at both the ends of the data points. Each test is represented by n > 20 data points, and each dot represents an average of n > 30 siliques per test. One-way ANOVA P value > 0.05 for panels D and E.

Traditional histological methods for visualizing *Arabidopsis* siliques employ organic solvents and typically require > 24 h before image capture. Our new silique imaging tool boasts minimal sample processing and faster experimental turnaround time, through the use of X-ray imaging. The X-ray images in Figure 3 and the measurements shown in Figure 4 show the suitability and reproducibility of using X-ray imaging for visualizing *Arabidopsis* siliques at different stages of development. We originally imaged only single siliques per X-ray exposure; however, the field of view of our setup allows for at least four siliques and a scale bar to be imaged per X-ray exposure at the highest usable image resolution (Figure 3). This feature allows for higher throughput and reducing variability between experiments.

The results of our three independent experiments indicate that the seed count and diameter are consistently reproducible between experiments (Figure 4). In summary, the custom X-ray imaging system offers significant advantages over traditional silique and seed visualization methods relying on clearing/fixative reagents. It is envisioned that researchers may adapt this new tool for imaging silique and seed development of different plant species.

## Validation of protocol

This protocol is validated by providing data to support the claims. Please refer to Figure 4, panel D & E, for details on the number of replicates and statistical tests.

## Notes

In our experiments, we did not find any phenotypic changes in the *Arabidopsis* plants cultivated from seeds that were exposed to X-rays.
